# Putatively Identified Sarmentoside-B Removes Oligomerized Amyloid Peptide from Neurons by Inhibiting mTOR and Restoring Lysosomal Function, in In Vitro Alzheimer’s Disease Model

**DOI:** 10.3390/pharmaceutics18060696

**Published:** 2026-06-04

**Authors:** Bruna Rojas Fróes, Juliana Guanaes Pina, Mariana da Mata Alves, Alquiandra S. F. Mançano, Fernanda C. Cardoso, Juliana Mozer Sciani

**Affiliations:** 1 Laboratório de Produtos Naturais, Universidade São Francisco, Bragança Paulista 12916-900, SP, Brazil; bruna.froes@usf.edu.br (B.R.F.); juliana.pina@mail.usf.edu.br (J.G.P.); alquiandra.mancano@mail.usf.edu.br (A.S.F.M.); 2 Laboratório de Bioquímica e Biofísica, Instituto Butantan, São Paulo 05503-900, SP, Brazil; m.alves.proppg@proppg.butantan.gov.br; 3 Centre for Motor Neuron Disease Research, Faculty of Health, Medicine and Behavioural Sciences, Institute for Molecular Biosciences, The University of Queensland, Brisbane, QLD 4072, Australia; f.cardoso@uq.edu.au

**Keywords:** Alzheimer’s disease, *Eudendrium carneum*, sarmentoside B, autophagy, lysosome, amyloid plaque clearance

## Abstract

**Background/Objectives**: Alzheimer’s disease (AD) is characterized by beta-amyloid (Aβ) plaque deposition, which impairs several cellular processes, including autophagy. Considering the multifactorial nature of AD, the development of therapies acting on alternative molecular targets is necessary. In this study, we evaluated the neuroprotective effect of a molecule from the hydrozoan *Eudendrium carneum* and investigated its impact on autophagy-related pathways. **Methods**: The secretion of *E. carneum* was fractionated by RP-HPLC according to its neuroprotective activity in SH-SY5Y cells exposed to oAβ42, evaluated using LDH and MTT assays. The purified molecule (named EC5), characterized by mass spectrometry, was evaluated regarding *in silico* toxicity and calcium dynamics. Neuronal lysosomal morphology was assessed using the LysoTracker probe, and cathepsin D activity was determined using a synthetic substrate. The expression of autophagy-related proteins (mTOR, LAMP-1, and LC3B) was evaluated by dot blotting, and amyloid plaque clearance was quantified using Thioflavin-T staining. **Results**: The steroid glycoside putatively identified as Sarmentoside B (EC5) exhibited neuroprotective effects and showed no toxicity or alterations in neuronal calcium or sodium channel dynamics. EC5 restored lysosomal morphology and cathepsin D activity, reversing the impairment induced by oAβ42. Furthermore, EC5 reduced mTOR expression, and this interaction was supported by molecular docking analysis. Lysosomal restoration promoted the clearance of oAβ42 aggregates, as evidenced by Thioflavin-T staining, resulting in reduced neuronal death. **Conclusions**: EC5, putatively identified as Sarmentoside B, exerts neuroprotective effects against oAβ42-induced toxicity by promoting autophagy-related amyloid clearance, highlighting its therapeutic potential for AD.

## 1. Introduction

Alzheimer’s disease (AD) is a progressive neurodegenerative disorder characterized by synaptic loss, neuronal degeneration, and cognitive impairment [1]. Among its main pathological features are the extracellular accumulation of beta-amyloid peptide (Aβ) and the formation of intraneuronal aggregates of hyperphosphorylated tau protein, events that trigger cellular stress, inflammation, and neuronal death [2,3]. The formation and aggregation of Aβ resulting from the abnormal processing of amyloid precursor protein (APP) are considered central events in the pathophysiology of AD. This peptide can accumulate either extracellularly or intracellularly in neurons, where its soluble oligomers exert early neurotoxic effects [1,4].

Beyond protein accumulation, dysfunction of autophagy pathways has emerged as a key factor in disease progression. Autophagy is an essential process involved in the degradation of damaged or misfolded, or aggregated proteins, ensuring the maintenance of cellular homeostasis, especially in neurons, in which proteostasis pathways are critical [5].

Amyloid aggregates are encapsulated by autophagic vesicles and addressed to lysosomes, which contain proteolytic enzymes responsible for protein degradation. In AD, the autophagy pathway is impaired, leading to the accumulation of immature autophagic vacuoles and hyperactivation of the mTOR signaling pathway. These events reduce autophagic flux and impair Aβ clearance, thereby contributing to neuronal toxicity [6].

In this context, there is growing interest in the identification of new molecules capable of modulating cellular pathways associated with autophagy restoration in AD [7]. To achieve this goal, the marine environment, due to its enormous biodiversity and chemical diversity, emerges as a strategic source for the discovery of bioactive compounds [8]. Our research group has previously isolated several molecules from marine animals exhibiting antioxidant, anti-inflammatory, beta-secretase inhibitory, and anti-amyloid aggregation activities, as well as the ability to reduce amyloid peptide-induced neuronal death [9,10,11,12,13,14].

Previous studies from our group demonstrated that the secretion of the hydrozoan *Eudendrium carneum* reduces oAβ42-induced cell death, suggesting the presence of active compounds with therapeutic potential for AD. Therefore, in the present study, we aimed to isolate and characterize an active molecule from *E. carneum* capable of reducing amyloid peptide-induced neuronal death and to investigate the molecular mechanisms underlying these effects, with emphasis on the autophagic pathway and lysosomal restoration following oAβ42 exposure.

## 2. Materials and Methods

### 2.1. Samples

Specimens of *E. carneum* were collected in São Sebastião/SP, under authorization from ICMBio (93947-1), and the extract was obtained as described by Moreno et al. [9]. Briefly, the animal was immersed in a solution of methanol containing 0.1% acetic acid for 48 h at room temperature. The secreted content, diluted in this solution, was centrifuged at 5000× *g* for 10 min and the supernatant was lyophilized and stored at −20 °C. This extract was resuspended in ultrapure water containing 0.1% TFA and subjected to fractionation by reverse-phase high-performance liquid chromatography (RP-HPLC) using a C-18 column (4.6 × 250 mm, 5 Å Phenomenex, Torrance, CA, USA). Elution was performed at a constant flow rate of 1 mL·min^−1^ using a 30 min gradient with solvents A (ultrapure water containing 0.1% trifluoroacetic acid) and B (90% acetonitrile in ultrapure water containing 0.1% trifluoroacetic acid). Peaks were detected at λ = 214 nm and collected manually according to the peak profile. The obtained fractions were tested in neuron cultures (described below) and active peaks were analyzed by ESI-MS/MS mass spectrometry (IT-TOF, Shimadzu Co, Kyoto, Japan) for purity assessment and characterization.

For characterization, mass spectrometry analysis was performed in both positive and negative modes over a scan range of 50–1500 *m*/*z*. The main ion was fragmented by collision with argon gas (50%), and the resulting fragments were manually analyzed to verify functional groups, as well as submitted to database searches to check for similarity with other molecules described in natural products. Raw files were converted to mzML using MSConvert (ProteoWizard 3.0) and processed through the GNPS-MassIVE public data repository for untargeted MS2 data using compound identification and molecular networking based on MS2 and spectral similarity (https://gnps.ucsd.edu/). Data parameters were set with a precursor ion mass tolerance of 0.5 Da, fragment ion mass tolerance of 0.2 Da, combined peaks of 6 min, and a threshold of 0.7. All public GNPS spectra curated by the natural products scientific community were utilized.

### 2.2. Neuronal Culture

SH-SY5Y cells (European Collection of Authenticated Cell Culture, Salisbury, UK) were cultured in DMEM/F12 medium containing 10% fetal bovine serum and 1% penicillin/streptomycin, maintained at 37 °C in a humidified atmosphere with 5% CO_2_.

After growth, cells were transferred to 96-well plates at 5 × 10^3^ cells/well. To induce the Alzheimer’s disease cellular model, SH-SY5Y cells were treated with synthetic oligomerized Aβ42 peptide (5 μM, GenScript, Piscataway, NJ, USA) for 48 h, following the method established by the group in previous studies [12].

The molecules obtained after chromatography (lyophilized and diluted in sterile PBS) were incubated with the cells at a concentration of 10 μg·mL^−1^ for 24 h. After this period, neurons were observed under light microscopy for general morphological assessment and evaluated as described in the following sections.

For all experiments, three groups were considered:

− Cells without any treatment;
− Cells exposed to oAβ42;
− Cells exposed to oAβ42 and subsequently treated with the molecule.

Each analysis was performed in triplicate, in two independent experiments, and all values were considered in the calculation.

### 2.3. Cell Viability

The MTT (3-(4,5-Dimethylthiazol-2-yl)-2,5-Diphenyltetrazolium Bromide) assay was employed as a colorimetric method to assess cell viability. After treatment, the reagent was added to the cells (0.5 mg·mL^−1^) and after 3 h in the 37 °C incubator, the formazan crystals were diluted in DMSO and the absorbance read in λ = 570 nm.

Alternatively, cell viability was assessed by quantifying LDH (lactate dehydrogenase), which evaluates plasma membrane integrity following apoptosis or necrosis. For this, 10 μL of the cell culture medium, after treatment, was mixed with 60 μL of reagent (LDH Cytotoxicity Assay Kit, Promega, WI, USA) in a 96-well plate. Luminescence was read after 30 min at room temperature.

The percentage of cytotoxicity was calculated considering the negative control as 100% viable cells.

### 2.4. Lysosome Evaluation

To assess lysosomal functionality after amyloid peptide exposure and treatment with the molecules, neurons were incubated with Lysotracker Green (Invitrogen™, Waltham, MA, USA), which labels acidic organelles, such as lysosomes. Treated cells were trypsinized, incubated with the probe (25 nM), and kept at 37 °C for 30 min. Cells were analyzed by flow cytometry, acquiring 10,000 events (Guava^®^ easyCyte, Millipore, Burlington, MA, USA). An initial gate was determined with non-labeled or treated cells (R1), being control for treatment and labeling. After that, we analyzed cells labeled, but without treatment, and cells treated with oAβ42 or oAβ42 + EC5, using the same gate set previously. The calculation of % of cells in R1 and R2 (cells positioned outside R1) was calculated using guavaSoft™ 2.7 software.

Additionally, treated cells were immersed in lysis buffer (sodium citrate buffer, pH 4.5, containing 1 mM EDTA, 2 mM PMSF, and 1 mM DTT) and submitted to ultrasonic pulses (3 pulses of 10 s), followed by centrifugation at 11,180× *g* for 15 min at 4 °C. The supernatant had the protein concentration determined by Bradford assay, and 10 μg of protein was incubated with a synthetic substrate, specific for cathepsin D (Bz-Arg-Gly-Phe-Phe-Pro-4M2NA, Sigma-Aldrich, Burlington, MA, USA), in sodium citrate buffer pH 4.0. Fluorescent values were obtained in λex = 365 nm and λem = 410–460 nm each 10 min, until 60 min, at 37 °C.

### 2.5. Protein Expression Evaluation

Anti-LC3B (PA146286, Invitrogen™, Waltham, MA, USA), Anti-LAMP-1 (MA529385, Invitrogen™, Waltham, MA, USA), and anti-mTOR (PA534663, Invitrogen™, Waltham, MA, USA) was used to identify autophagy-related proteins in control or treated neurons by dot blot. Ten microliters of sample (1 mg·mL^−1^) was deposited in a nitrocellulose membrane, which was positioned in the SNAP i.d™ System (Millipore, Burlington, MA, USA). BSA 1% was added for blockage and then the primary antibody was incubated (all of them 1:1000). After wash with TBS-T, the secondary antibody was incubated (1:1000, 32460, Invitrogen) and the detection was made using the ECL Substrate (Pierce™ Fast Western Blot Kit, Thermo Scientific™, Waltham, MA, USA).

Images were acquired by ChemiDoc™ Imaging System (Biorad, Hercules, CA, USA) and spots were measured using ImageJ software (v1.54) to determine area of plots, identified automatically, in triplicate.

### 2.6. Molecular Docking

The mTORc1 (target protein) was evaluated in two models: 3JBZ and 2FAP from PDB (Protein Data Bank), chosen based on their resolution by the X-ray diffraction method (~2 Å) and isolated from humans. PDB codes were individually inserted in SwissDock platform and AutoDock Vina mechanism was selected. The search space was defined as 20 × 20 × 20 Å box size and box center −5x −35x −39 for 3JBZ and −11 × 25 × 35 for 2FAP (in the X-, Y-, and Z-axes, respectively), place defined by the analysis of Cavities, by UCSF ChimeraX v1.10, and also by the literature, considering relevant amino acids for the biological activity of the proteins.

The ligand was inserted in SwissDock by its SMILES code provided by PubChem. Analysis of bind (location, energy, and distances between atoms) was performed using UCSF ChimeraX v1.10.

### 2.7. Assessment of Amyloid-β42 Clearance

To quantify oAβ42 clearance, samples of the supernatant were analyzed using the ThT assay. An aliquot of 10 μL of supernatant of the culture was incubated with 5 μL of ThT (1 mM diluted in 50 mM PBS pH 7.4) and 5 μL of PBS, and after 10 min of incubation at room temperature, in the dark, fluorescence was measured at λex = 365 nm and λem = 410–460 nm.

### 2.8. Calcium Dynamics

SH-SY5Y cells, cultured according to the method described above, were plated at 40,000 cells per well in 384-well flat clear-bottom black plates (Corning Inc., Corning, NY, USA) and maintained at 37 °C in a humidified 5% CO_2_ incubator for 48 h before assaying as previously described by us [15]. Briefly, after 48 h incubation, cells were incubated with Calcium 4 dye (Molecular Devices, Sunnyvale, CA, USA) reconstituted in assay buffer containing (in millimolar) 140 NaCl, 11.5 glucose, 5.9 KCl, 1.4 MgCl_2_, 1.2 NaH_2_PO_4_, 5 NaHCO_3_, 1.8 CaCl_2_, and 10 HEPES (pH 7.4) and incubated for 30 min at 37 °C in a humidified 5% CO_2_ incubator. Fluorescence responses were recorded using excitation of 470 nm and emission of 515 nm for 10 s to set the baseline, and then 600 s after incubation of EC5 (1st addition) and for a further 600 s after incubation of 90 mM KCl + 5 mM CaCl_2_ or 50 μM veratridine (2nd addition).

### 2.9. In Silico Toxicity

The toxicity of Sarmentoside B was predicted using ProTox 3.0—Prediction of Toxicity of Chemicals. The structure of the molecule was drawn, according to PubChem, and the report containing the potential toxicity was generated, with default parameters.

### 2.10. Statistical Analysis

Results were processed using GraphPad Prism software (v.10) to calculate mean ± SEM from data. Data were plotted in graphs for bar chart generation and group comparison. The Shapiro–Wilk test confirmed the normality of data, which indicated the application of ANOVA test. Thus, difference between groups was evaluated using one-way ANOVA followed by Tukey’s post hoc test, considering *p* < 0.05.

## 3. Results

### 3.1. Fractionation of the E. carneum Extract

**Figure 1:**
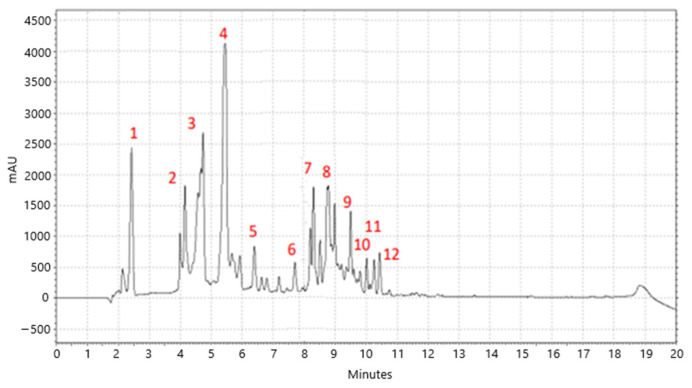
Chromatogram of methanolic extract of *E. carneum*, obtained after reverse-phase high-performance liquid chromatography (RP-HPLC). Numbers indicate the fractions manually collected and used in the biological assays.

The secretion obtained from *E. carneum* was subjected to reverse-phase high-performance liquid chromatography (RP-HPLC), resulting in the separation of 12 distinct fractions, which were manually collected and named EC1 to EC12 (Figure 1). For collection, only the most relevant and intense chromatographic peaks were selected, whereas less prominent peaks, although visually detectable, were not collected.

### 3.2. Cell Viability After EC5 Treatment

**Figure 2:**
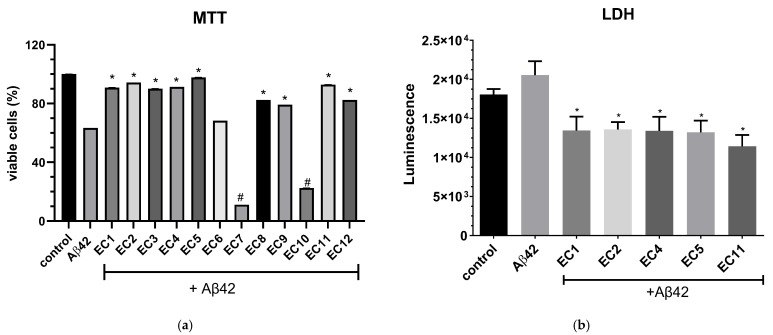
Viability of SH-SY5Y cells without treatment (control), oAβ42, or *E. carneum* HPLC fractions. (**a**) Evaluation by MTT assay; (**b**) evaluation by LDH assay. Data are presented as mean ± SEM. * *p* < 0.001 vs. Aβ42, showing a neuroprotective effect; # *p* < 0.001 vs. Aβ42, showing a neurotoxic effect.

The 12 HPLC fractions were evaluated using two cell viability assays in an in vitro model of AD. In the MTT assay, treatment with oAβ42 reduced cell viability to approximately 60% compared to the control group (Figure 2a). Treatment with EC1, EC2, EC3, EC4, EC5, EC11, and EC12, after oAβ42 exposure, increased cell viability to approximately 90%, with EC5 reaching 97.7%, similar to the control group. This assay enabled the identification of the fractions that best preserved cell viability, with EC5 and EC11 showing the highest protective effects. In contrast, fractions EC7 and EC10 exhibited the lowest viability indices, with values even lower than those observed in the oAβ42-treated group.

To confirm these findings, the lactate dehydrogenase (LDH) release assay was performed using the five fractions with the best performance in the MTT assay (EC1, EC2, EC4, EC5, and EC11). All evaluated fractions promoted increased cell viability after treatment (Figure 2b).

### 3.3. Molecular Structure Analysis

**Figure 3:**
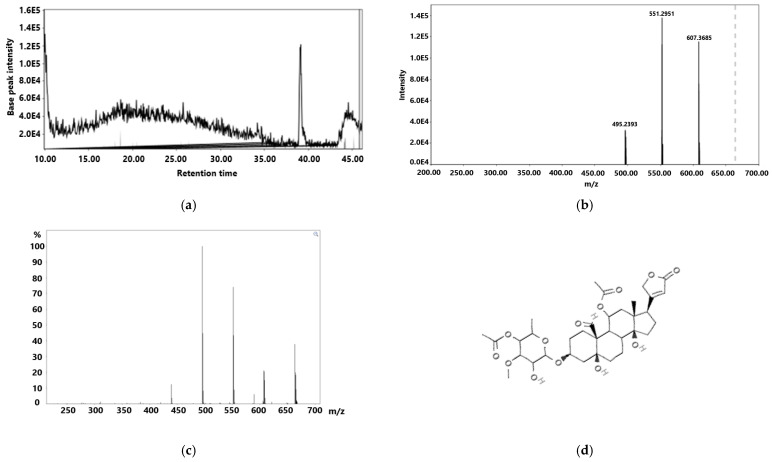
Characterization of EC5 active fraction. (**a**) TIC spectra of EC5 fraction, obtained from HPLC, in an MS scan, in positive ionization mode. The peak is correspondent to EC5; (**b**) MS/MS spectra of EC5, after fragmentation of 663 *m*/*z* ion. (**c**) MS/MS spectra of Sarmentoside B deposited in the GNPS database, showing the same fragmentation pattern as experimental MS/MS (shown in Figure 3b). (**d**) Chemical structure of Sarmentoside B from PubChem.

According to the mass spectrometry analysis, a purified biologically active molecule was isolated from fraction EC5, which showed the best results in the MTT assay and was further confirmed by the LDH assay. This molecule corresponds to 4% of the secretion, calculated based on dry weight of all collected peaks. Initial characterization was performed by mass spectrometry in positive ionization mode, and the major ion (*m*/*z* 663) was selected for fragmentation analysis (Figure 3a,b).

To further investigate the structure, the raw data were submitted to the GNPS platform, which uses spectral similarity networks to compare experimental spectra with those available in its database (Figure 3c). This analysis revealed similarities in mass and fragmentation patterns with Sarmentoside B (Figure 3d), within the predefined tolerance range. Furthermore, the fragmentation profile of EC5 was manually analyzed and compared with the literature and database information regarding Sarmentoside B fragmentation, indicating this molecule as a possible candidate. Therefore, EC5 was putatively identified as Sarmentoside B.

### 3.4. Lysosome Evaluation and Autophagy-Related Proteins

**Figure 4:**
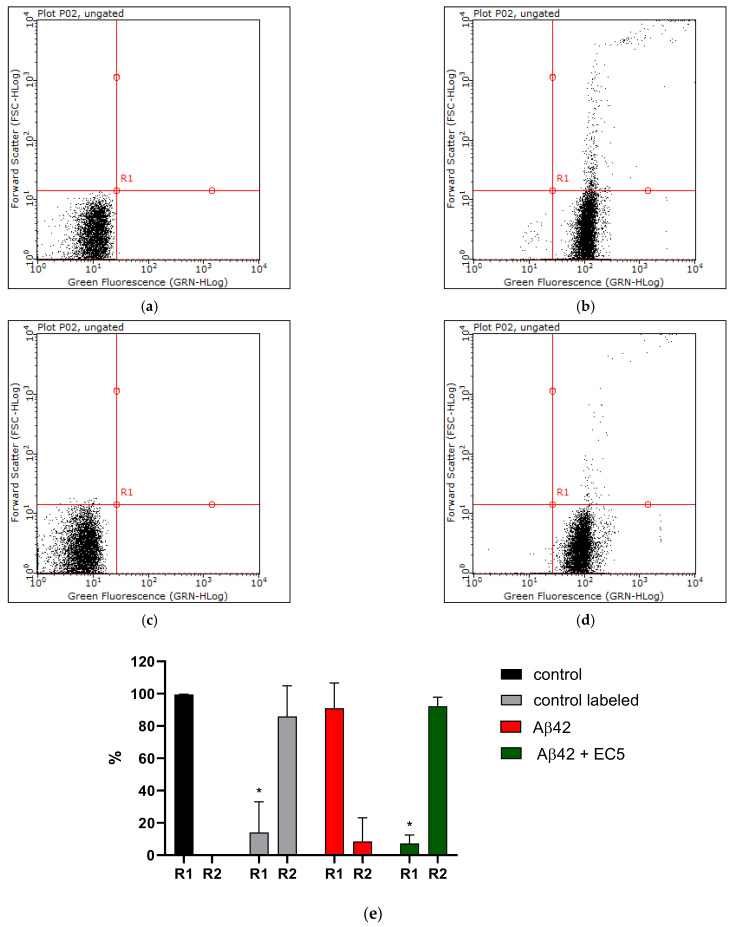
SH-SY5Y labeled with Lysotracker probe after being treated with oAβ42 or EC5. (**a**) Cells without labeling or treatment, used for gate R1 setting; (**b**) control cells, labeled with lysotracker, but without any treatment; (**c**) labeled cells treated with oAβ42; (**d**) labeled cells treated with oAβ42 and EC5; (**e**) percent of R1 (gate determined with cells without labeling) and R2 (gate for cells labeled with Lysotracker) as mean ± SEM. * *p* < 0.001 for R1 analysis vs. oAβ42.

Lysosomal integrity was evaluated using the fluorescent LysoTracker probe followed by flow cytometry analysis. Initially, untreated and unlabeled neurons were analyzed to establish the gating strategy (Figure 4a). Control neurons labeled with the probe, but without treatment, were positioned to the right of the previously established gate, indicating lysosomal labeling and preserved organelle integrity (Figure 4b). In contrast, treatment with 5 μM oAβ42 reduced fluorescence to levels similar to those observed in unlabeled cells, indicating lysosomal disruption (Figure 4c). Treatment with EC5 (10 μg·mL^−1^) restored lysosomal labeling to levels comparable to the control group (Figure 4d). Quantitative analysis of the percentage of events in gates R1 and R2 further demonstrated the protective effect of EC5 on lysosomal integrity (Figure 4e).

**Figure 5:**
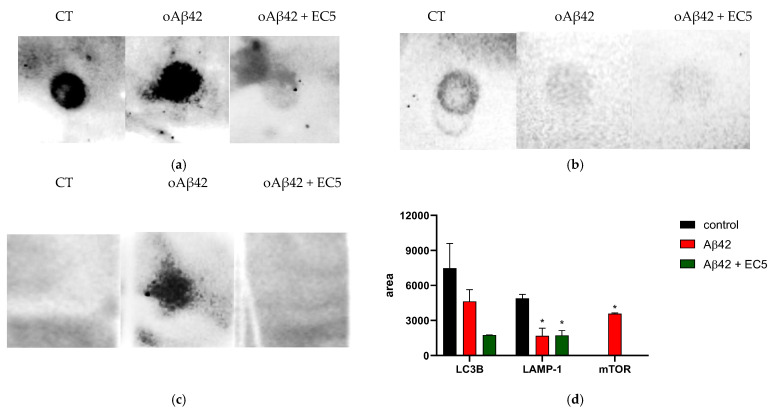
Dot blot of autophagy-related proteins obtained from SH-SY5Y after treatment with oAβ42 or oAβ42 + EC5 (10 μg·mL^−1^). (**a**) Anti-LC3B; (**b**) anti-LAMP-1; (**c**) anti-mTOR; (**d**) area of the spots presented as mean ± SEM. * *p* < 0.005 vs. control group.

Autophagy-related proteins, including mTOR, LC3B, and LAMP-1, were evaluated in treated neurons. LC3B and LAMP-1 levels were reduced after oAβ42 exposure compared to the control group (Figure 5a,b, respectively), although the reduction in LC3B was not statistically significant (Figure 5d). Treatment with EC5 at 10 μg·mL^−1^ did not alter the expression of either protein. In contrast, mTOR expression increased following oAβ42 exposure and was reduced after EC5 treatment (10 μg·mL^−1^; Figure 5c).

When lower concentrations of EC5 were tested, a concentration-dependent response was observed. No detectable inhibition was observed at 0.1 μg·mL^−1^, whereas inhibition was detected at 1 μg·mL^−1^, although less pronounced than at 10 μg·mL^−1^ (Figure S1, Supplementary Materials).

**Figure 6:**
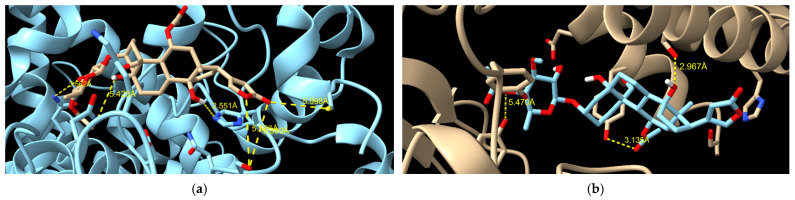
Molecular docking to show interaction between Sarmentoside B and mTORc1. Yellow dashes show the interaction and atom distance of two parts of mTOR: (**a**) 3JBZ and (**b**) 2FAP.

To further investigate the interaction between EC5 and mTOR, molecular docking analysis was performed using two protein models: 3JBZ, corresponding to the ATP/ADP binding site, and 2FAP, corresponding to the rapamycin-binding site. EC5 interacted with 3JBZ with a binding affinity of −6.67 kcal·mol^−1^ (Table 1 and Figure 6a), involving interactions with Gln2167, a key residue for ATP binding, and His2340, located near Asp2338, the catalytic site of the protein. Regarding the 2FAP model, EC5 exhibited a binding affinity of −9.832 kcal·mol^−1^ and occupied a similar region to rapamycin, interacting with Ser2035, near the rapamycin-associated residue Glu2032 (Table 1 and Figure 6b). These findings suggest a potential interaction between EC5 and mTOR.

### 3.5. Cathepsin D Activity

**Figure 7:**
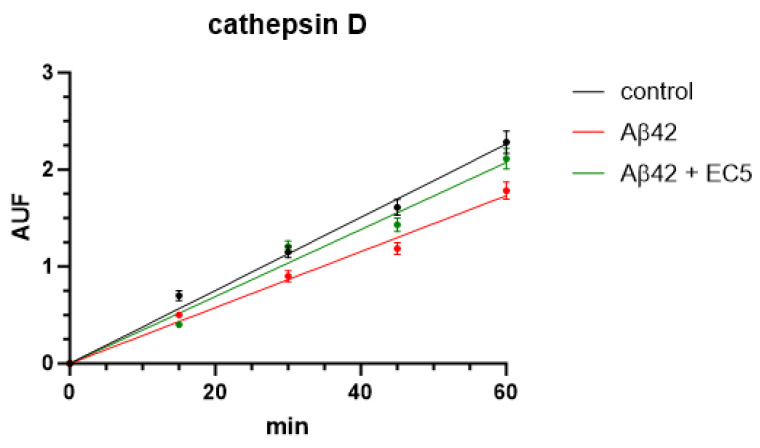
Catalytic activity of cathepsin D from SH-SY5Y neurons, determined using specific synthetic substrate, after exposure to oAβ42 and oAβ42 + EC5. Arbitrary units of fluorescence (AUF) over time, showing the mean ± SEM of each point.

Cathepsin D activity in neuronal lysates was evaluated using a specific fluorogenic substrate. The control group exhibited the highest fluorescence increase and reaction velocity. In contrast, neurons treated with 5 μM oAβ42 showed reduced reaction velocity, suggesting decreased cathepsin D activity and lysosomal dysfunction, consistent with the toxic effects of the amyloid peptide. The group treated with oAβ42 followed by EC5 (10 μg·mL^−1^) exhibited intermediate fluorescence values, significantly higher than those observed in the oAβ42 group, indicating partial preservation of cathepsin D activity (Figure 7).

### 3.6. Assessment of Protein Aggregate Removal

**Figure 8:**
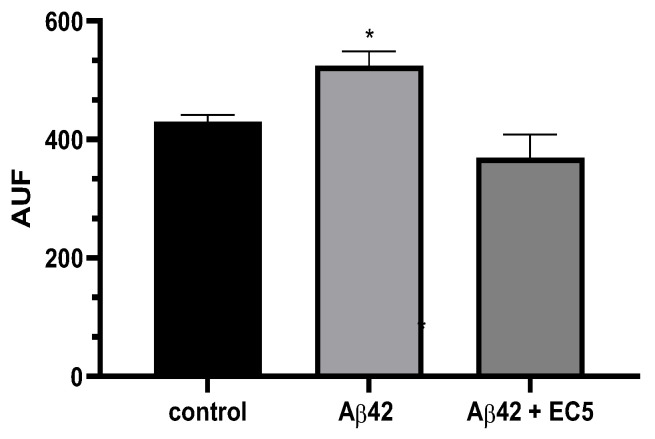
Arbitrary units of fluorescence (AUF) of protein aggregates from SH-SY5Y neurons by Thioflavin-T labeling, in three experimental groups: control, oAβ42, and oAβ42 + EC5. * *p* < 0.05 vs. control.

Protein aggregates were quantified using the Thioflavin-T (ThT) assay following treatment with oAβ42 or oAβ42 + EC5. Comparison among the groups revealed differences in fluorescence intensity (Figure 8). Neurons treated with oAβ42 showed increased levels of ThT-positive aggregates, whereas treatment with EC5 reduced aggregate levels to values similar to those observed in the control group. These findings indicate that EC5 reduced the amount of oAβ42 aggregates.

### 3.7. Calcium Homeostasis

**Figure 9:**
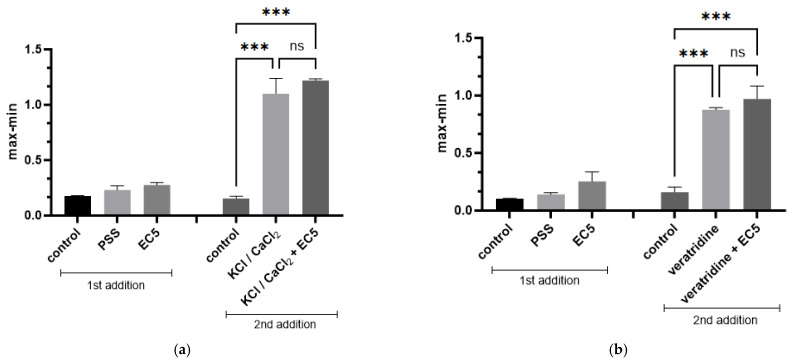
Maximum over minimum response (max–min) of fluorescence inside SH-SY5Y neurons, detected by calcium probe, after stimulation of (**a**) calcium channels, by KCl/CaCl_2_, and (**b**) sodium channels, by veratridine, in the absence or presence of EC5. *** *p* < 0.001 vs. control; ns = not significant.

To evaluate whether EC5 affects neuronal calcium homeostasis, intracellular calcium dynamics were analyzed using a fluorescent probe following stimulation of calcium and sodium channels with KCl/CaCl_2_ and veratridine, respectively. EC5 (10 μg·mL^−1^) did not alter basal calcium levels in SH-SY5Y neurons (Figure 9a,b, first addition). After addition of the channel agonists, intracellular calcium levels increased in response to KCl/CaCl_2_ stimulation of calcium channels (Figure 9a, second addition) and veratridine stimulation of sodium channels (Figure 9b, second addition). EC5 neither inhibited nor potentiated these responses, indicating that the molecule did not interfere with ion channel activity or calcium dynamics in neurons.

### 3.8. Predicted Toxicity

Potential toxic effects of Sarmentoside B were predicted using in silico approaches. Several targets related to organ toxicity, receptors, metabolic pathways, and cellular processes were analyzed. Possible nephrotoxicity and respiratory toxicity were predicted with low probability, as well as potential interactions with androgen and estrogen receptors, aromatase, acetylcholinesterase (AChE), p53, NADH-quinone oxidoreductase, and PRX (Table 2). In contrast, immunotoxicity, nutritional toxicity, and mitochondrial membrane potential (MMP) modulation were identified as potentially relevant targets.

## 4. Discussion

Autophagy is an essential process for maintaining proteostasis in neurons, and its dysfunction has been strongly associated with AD progression. Therefore, molecules capable of restoring autophagic activity may represent an alternative strategy to promote amyloid clearance and improve disease-related alterations [16].

Based on previous studies from our group demonstrating that the secretion of *E. carneum* reduces amyloid peptide-induced neuronal death, we isolated an active molecule using bioassay-guided fractionation. The main criterion for fraction selection was the reduction of neuronal death, followed by the evaluation of lysosomal function [6].

Two complementary methods were used to evaluate cell viability: MTT and LDH assays. The MTT assay measures cellular metabolic activity through the reduction of the yellow tetrazolium salt MTT into insoluble purple formazan crystals by mitochondrial dehydrogenases in viable cells [17]. In contrast, the LDH assay evaluates membrane integrity by detecting lactate dehydrogenase released into the extracellular medium after membrane damage, serving as an indirect indicator of cell death [18].

Using activity-guided fractionation, we putatively identified Sarmentoside B through tandem mass spectrometry (MS/MS) analysis combined with spectral comparison using the GNPS database. Sarmentoside B was originally isolated from *Strophanthus sarmentosus* seeds [19], but has also been reported in other plant species, including *Annona muricata* L. [20]. More recently, studies have suggested that Sarmentoside B may actually be synthesized by bacteria associated with plants and animals, including soil-derived *Streptomyces cavourensis* [21], and bacteria from the human oral microbiome [22]. In marine environments, Sarmentoside B has been identified in the brown seaweed *Sargassum duplicatum* [23] and in bacteria associated with sea cucumbers [21], using extraction and analytical approaches similar to those employed in the present study. Importantly, these studies also used methanolic extraction and reported retention times comparable with those observed here.

Sarmentoside B is a cardenolide glycoside with the molecular formula C_34_H_48_O_13_. Cardenolide glycosides are commonly found in plants as defense molecules against herbivores and have also been identified in animals, particularly amphibians [24]. In the Bufonidae family, bufadienolides have been widely investigated and used in traditional Asian medicine preparations such as Ch’an Su for the treatment of several diseases [25].

The presence of multiple hydroxyl, methoxy, and ester groups confers physicochemical properties that may influence both bioavailability and binding affinity to molecular targets [26]. Biologically, Sarmentoside B shares structural characteristics with cardiotonic glycosides, which are classically described as inhibitors of Na^+^/K^+^-ATPase. Inhibition of this transporter results in increased intracellular sodium concentrations and consequent elevation of intracellular calcium through the Na^+^/Ca^2+^ exchanger, enhancing myocardial contractility [27,28,29].

Potential toxic effects related to this mechanism were not supported by our analyses. In silico toxicity prediction did not indicate cardiotoxicity, and calcium dynamics experiments showed that EC5 neither activated nor inhibited calcium or sodium channels, nor altered calcium mobilization in neurons. The main predicted concern was immunotoxicity, detected with relatively high probability in silico. In general, cardiac glycosides are not considered classical immunosuppressive agents, but may modulate immune signaling through Na^+^/K^+^-ATPase-dependent activation of Src/MAPK/NF-κB pathways, thereby influencing cytokine production, inflammatory responses, and immune cell activation [30]. However, our findings suggest that Sarmentoside B may not strongly activate Na^+^/K^+^-ATPase-associated signaling. Furthermore, low concentrations of cardiac glycosides, such as ouabain, have been reported to exert anti-inflammatory effects. Therefore, additional studies are necessary to better understand the immunological implications of Sarmentoside B and whether these effects may be relevant for future therapeutic applications.

Beyond their cardiotonic properties, cardenolides have also been reported to exhibit anti-inflammatory, antioxidant, and cell-signaling modulatory activities, supporting their potential pharmacological applications [31].

Cardiac glycosides have been described as modulators of autophagy not only in cancer cells, but also in neuronal models, in which controlled autophagy induction may contribute to neuroprotection and aggregate clearance [32,33]. Studies using AD models have demonstrated promising effects of cardenolide glycosides in neurodegeneration. Thakur et al. isolated three cardenolide glycosides through bioassay-guided fractionation and demonstrated their ability to reduce Aβ42 levels in cell culture models [34]. Moreover, several cardiac glycosides, including bufalin, digitoxin, ouabain, peruvoside, convallatoxin, and proscillaridin, activate AMPK, which subsequently inhibits mTOR and stimulates autophagy, but focused on other diseases [32].

Digoxin reduced TNF-α levels and restored choline acetyltransferase activity, improving memory and neuronal survival in rats [35]. Similarly, ouabain improved cognitive function in FAD4T transgenic mice through TREM2 upregulation and PI3K/Akt pathway activation, resulting in anti-inflammatory effects [36]. Furthermore, ouabain reduced tau pathology in mouse models and modulated the autophagic pathway by promoting transcription factor EB activation through mTOR inhibition [37].

In the present study, EC5 reduced mTOR expression, which had previously been increased following oAβ42 treatment. Molecular docking analyses using the 3JBZ and 2FAP models further suggested interactions between Sarmentoside B and mTOR. These models were selected because they represent two important functional regions of the protein: the ATP/ADP binding site and the rapamycin-binding site, respectively. The stronger affinity observed for the 2FAP model, corresponding to the rapamycin-associated region, suggests a potential inhibitory interaction with mTOR [38].

It is well established that rapamycin induces neuronal autophagy through mTOR inhibition and consequent stimulation of autophagic flux [39]. Hyperactivation of the PI3K/Akt/mTOR pathway has been described in AD brains and is associated with impaired autophagy and increased protein aggregate accumulation [40]. In addition, studies in AD patients and experimental models have reported altered levels of autophagy markers, including LC3 [41].

However, as discussed by Rubinsztein and Nixon [39], autophagic activity cannot be inferred solely from LC3-II levels, since the protein may either accumulate or decrease depending on cell type and experimental conditions. Similar considerations apply to LAMP-1 expression. Therefore, although LC3B and LAMP-1 modulation was not clearly observed in our study, this does not necessarily exclude alterations in autophagic flux.

Thus, additional studies are required to better understand the mechanisms by which Sarmentoside B modulates autophagy, including direct evaluation of autophagic flux and analysis of mTOR phosphorylation status. Nevertheless, evaluation of the final stages of the autophagic process demonstrated relevant effects of Sarmentoside B on lysosomal morphology and functionality, which may explain the observed reduction in neuronal death.

Although no approved therapies currently target autophagy specifically for the treatment of AD, some drugs are being repositioned and evaluated in clinical trials. For example, metformin has been tested in patients with amnestic mild cognitive impairment, although no significant differences were observed in cognitive scores or plasma Aβ42 levels [42]. Similarly, rapamycin did not significantly alter the Global Clinical Dementia Rating Scale score in patients with mild cognitive impairment or early-stage dementia. However, the authors emphasized that further studies are necessary to better understand the clinical potential of rapamycin in AD before excluding its therapeutic applicability [43].

Lysosomal dysfunction in AD is associated with lysosomal membrane permeabilization, leading to the release of cathepsins into the cytosol. This process compromises cellular homeostasis, promotes protein aggregate accumulation, and may activate cell death pathways [44].

The fluorescent LysoTracker probe was used as an important tool to evaluate lysosomal integrity and functionality in neurons. Since this probe selectively accumulates in acidic organelles, fluorescence intensity can be used to assess lysosomal preservation [45]. Using this approach, we observed that EC5 preserved lysosomal labeling patterns similar to those observed in control cells.

In addition to lysosomal preservation, cathepsin D activity was restored after treatment with EC5. Cathepsin D is one of the main lysosomal proteases and plays an important role in the degradation of intracellular substrates, including misfolded proteins such as Aβ [46]. Under physiological conditions, this enzyme remains confined within lysosomes, where it contributes to protein turnover and aggregate clearance. Therefore, preservation of lysosomal morphology and restoration of cathepsin D activity suggest recovery of lysosomal functionality.

This restoration may explain the reduction in Thioflavin-T-positive aggregates observed after EC5 treatment. Thioflavin-T exhibits specific affinity for β-sheet-rich structures characteristic of amyloid aggregates, allowing indirect quantification of protein aggregation through fluorescence measurements [47]. Thus, although additional experiments must be performed using complementary techniques, such as immunocytochemistry or ELISA, we saw that the restoration of lysosomal morphology and proteolytic activity may have contributed to enhanced aggregate clearance.

Despite the promising findings, the present study was conducted exclusively in vitro, and additional experiments using other neuronal models and in vivo systems are necessary to confirm the efficacy of Sarmentoside B in promoting amyloid clearance through lysosomal restoration in more complex biological systems. Furthermore, animal studies will be important to evaluate the safety profile and pharmacokinetic properties of the molecule.

## 5. Conclusions

Sarmentoside B was able to restore lysosomal morphology and functionality and consequently reduce amyloid aggregates in neurons, one of the main pathological hallmarks of Alzheimer’s disease, thereby exerting a neuroprotective effect. Although the precise mechanisms of action still require further investigation, our findings suggest that the initiation of autophagy may be associated with its ability to modulate mTOR.

## Figures and Tables

**Table 1:** Molecular docking of Sarmentoside B and two models of mTORc1.

Protein	Binding Energy (kcal·mol^−1^)	Amino Acid Binding	Distance (Å)
3JBZ	−6.67	GLN 2167	5.423
		GLN 2194	3.562
		SER 2342	5.526 and 5.399
		CYS 2546	5.098
		HIS 2340	2.551
2FAP	−9.832	TYR 26	5.470
		SERFTa 2035	2.967
		TYR 2105	3.135

**Table 2:** Predicted toxicity of Sarmentoside B in organs and targets.

Target	Prediction	Probability
Hepatotoxicity	Inactive	0.94
Neurotoxicity	Inactive	0.92
Nephrotoxicity	Active	0.60
Respiratory toxicity	Active	0.78
Cardiotoxicity	Inactive	0.61
Carcinogenicity	Inactive	0.62
Immunotoxicity	Active	0.99
Mutagenicity	Inactive	0.89
Cytotoxicity	Inactive	0.96
BBB-barrier	Inactive	0.60
Ecotoxicity	Inactive	0.72
Clinical toxicity	Inactive	0.63
Nutritional toxicity	Active	0.98
Aryl hydrocarbon receptor	Inactive	1
Androgen receptor	Inactive	0.77
Androgen receptor ligand binding domain	Active	0.63
Aromatase	Active	0.83
Estrogen receptor alpha	Active	0.60
Estrogen receptor ligand binding domain	Inactive	0.99
Peroxisome proliferator activated receptor gamma	Inactive	0.62
Nuclear factor like 2/antioxidant responsive element	Inactive	0.93
Heat shock factor response element	Inactive	0.93
Mitochondrial membrane potential	Active	0.9
Phosphoprotein (tumor suppressor) p53	Active	0.59
ATPase family AAA domain-containing protein 5	Inactive	0.92
Thyroid hormone receptor alpha	Inactive	0.83
Thyroid hormone receptor beta	Inactive	0.95
Transtyretrin	Inactive	0.62
Ryanodine receptor	Inactive	0.74
GABA receptor	Inactive	0.56
Glutamate N-methyl-D-aspartate receptor	Inactive	0.99
alpha-amino-3-hydroxy-5-methyl-4-isoxazolepropionate receptor	Inactive	1
Kainate receptor	Inactive	1
Achetylcholinesterase	Active	0.50
Constitutive androstane receptor	Inactive	0.99
Pregnane X receptor	Active	0.68
NADH-quinone oxidoreductase	Active	0.57
Voltage gated sodium channel	Inactive	0.94
Na+/I- symporter	Inactive	0.60
Cytochrome CYP1A2	Inactive	0.99
Cytochrome CYP2C19	Inactive	0.96
Cytochrome CYP2C9	Inactive	0.84
Cytochrome CYP2D6	Inactive	0.90
Cytochrome CYP3A4	Inactive	0.98
Cytochrome CYP2E1	Inactive	0.99

## Data Availability

The raw data supporting the conclusions of this article will be made available by the authors on request.

## Supplementary Materials

The following supporting information can be downloaded at: https://www.mdpi.com/article/10.3390/pharmaceutics18060696/s1, Figure S1: Dot blot of proteins extracted from SH-SY5Y after treatment with oAβ42 or oAβ42 + EC5. (a) Anti-LC3B; (b) anti-LAMP-1; (c) anti-mTOR (upper panel 10 μg·mL^−1^ and lower panel 1 and 0.1 μg·mL^−1^). Yellow arrows show the control (CT), oAβ42, and oAβ42 + EC5 groups.

## Abbreviations

The following abbreviations are used in this manuscript:

Aβ	Amyloid beta-peptide
AD	Alzheimer’s Disease
APP	Amyloid precursor protein
EC	*Eudendrium carneum*
