# Supplementary material for: Putatively Identified Sarmentoside-B Removes Oligomerized Amyloid Peptide from Neurons by Inhibiting mTOR and Restoring Lysosomal Function, in In Vitro Alzheimer’s Disease Model
Source: Pharmaceutics. 2026 Jun 4;18(6):696. doi: 10.3390/pharmaceutics18060696 (PMC13305706; doi:10.3390/pharmaceutics18060696)
Supplement: Supplementary file 1 [file pharmaceutics-18-00696-s001.zip › pharmaceutics-4255178-supplementary.pdf]

Supplementary file 1

a

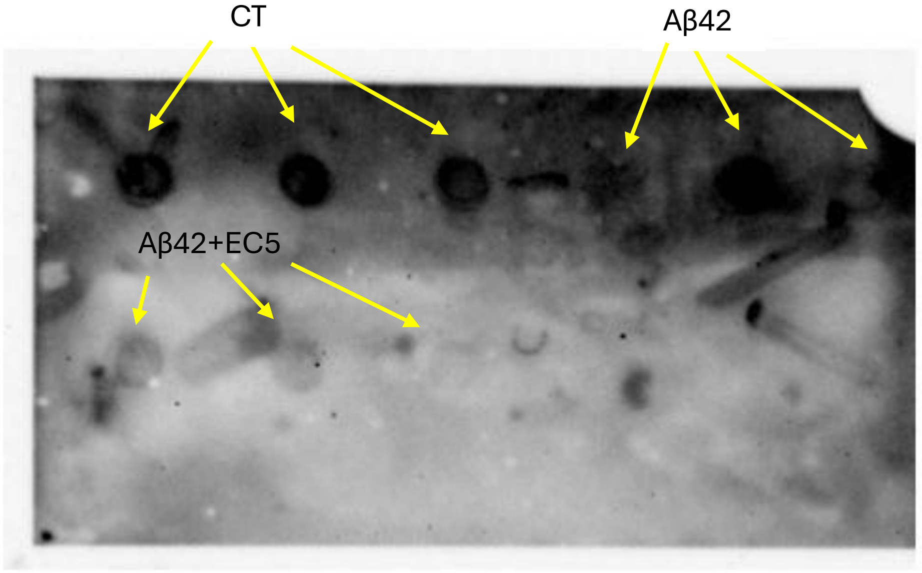

b

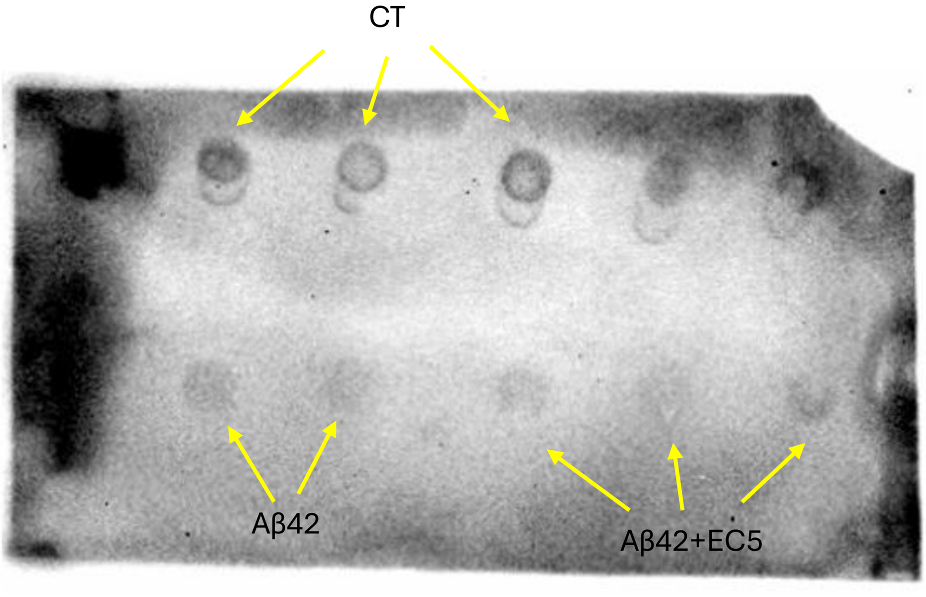

c

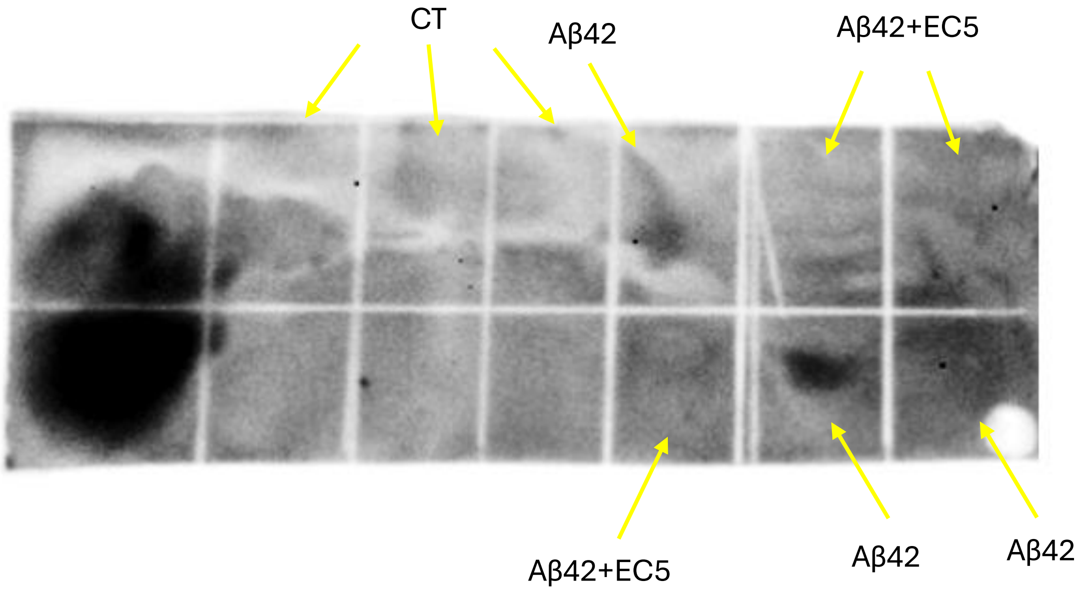

Dot blot of proteins extracted from SH-SY5Y after treatment with Aβ42 or Aβ42 + EC5. (a) anti-LC3B; (b) anti-LAMP-1; (c) anti-mTOR. Yellow arrows show the control (CT), Aβ42 and Aβ42 + EC5 groups.
